# Mechanical properties of the premature lung: From tissue deformation under load to mechanosensitivity of alveolar cells

**DOI:** 10.3389/fbioe.2022.964318

**Published:** 2022-09-16

**Authors:** Jonas Naumann, Nicklas Koppe, Ulrich H. Thome, Mandy Laube, Mareike Zink

**Affiliations:** ^1^ Research Group Biotechnology and Biomedicine, Peter-Debye-Institute for Soft Matter Physics, Leipzig University, Leipzig, Germany; ^2^ Center for Pediatric Research Leipzig, Department of Pediatrics, Division of Neonatology, Leipzig University, Leipzig, Germany

**Keywords:** fetal lung, lung mechanics, tissue deformation, alveolar epithelial cells, mechanosensitivity, epithelial sodium channel

## Abstract

Many preterm infants require mechanical ventilation as life-saving therapy. However, ventilation-induced overpressure can result in lung diseases. Considering the lung as a viscoelastic material, positive pressure inside the lung results in increased hydrostatic pressure and tissue compression. To elucidate the effect of positive pressure on lung tissue mechanics and cell behavior, we mimic the effect of overpressure by employing an uniaxial load onto fetal and adult rat lungs with different deformation rates. Additionally, tissue expansion during tidal breathing due to a negative intrathoracic pressure was addressed by uniaxial tension. We found a hyperelastic deformation behavior of fetal tissues under compression and tension with a remarkable strain stiffening. In contrast, adult lungs exhibited a similar response only during compression. Young’s moduli were always larger during tension compared to compression, while only during compression a strong deformation-rate dependency was found. In fact, fetal lung tissue under compression showed clear viscoelastic features even for small strains. Thus, we propose that the fetal lung is much more vulnerable during inflation by mechanical ventilation compared to normal inspiration. Electrophysiological experiments with different hydrostatic pressure gradients acting on primary fetal distal lung epithelial cells revealed that the activity of the epithelial sodium channel (ENaC) and the sodium-potassium pump (Na,K-ATPase) dropped during pressures of 30 cmH_2_O. Thus, pressures used during mechanical ventilation might impair alveolar fluid clearance important for normal lung function.

## Introduction

The mechanical properties of cells and tissues play a crucial role during embryogenesis in which cells differentiate and migrate, and morphogenesis leads to the formation of tissues and organs (Mammoto and Ingber, 2010). While most organs are already functioning in the fetal body *in utero*, the lung does not perform gas exchange, but rather works as an exocrine gland. In contrast, immediately after birth, it inflates with air to facilitate gas exchange between blood circulation and the external environment by continuous contraction and relaxation of respiratory muscles. Thus, the lung is never in a mechanically relaxed state. In fact, the mechanical behavior of the lung depends on the level of pre-stress provided by the transpulmonary pressure, as well as the tissue structure itself, dominated by the extracellular matrix (ECM) (Wirtz and Dobbs, 2000; Bates and Suki, 2008; Andrikakou et al., 2016).

While mechanical properties of lung tissue, single alveolar cells, as well as the ECM of the alveolar parenchyma have mainly been studied in adult lung tissue for decades (Alcaraz et al., 2003; Fredberg and Kamm, 2006; Faffe and Zin, 2009; Tschumperlin et al., 2010; Roan and Waters, 2011; Suki et al., 2011; Andrikakou et al., 2016; Knudsen and Ochs, 2018), much less is known about the vulnerable lung of preterm infants, who frequently suffer from respiratory distress syndrome (RDS), and its mechanical properties on length scales of the entire lung down to the cellular level (Knudsen and Ochs, 2018).

In healthy lungs of full-term infants and adults, the macroscopic and microscopic deformation behavior is mainly determined by surface tension effects at the alveolar air-liquid interface as long as volume changes and resulting strains are small (Wilson and Bachofen, 1982; Bachofen et al., 1987; Bachofen and Schürch, 2001; Knudsen and Ochs, 2018). At larger lung volumes, ECM components, mainly elastin and collagen (Suki et al., 2005, 2011), determine the mechanical properties of the lung and act as stress-bearing components to prevent overstretching and damage (Maksym and Bates, 1997; Suki et al., 2005; Faffe and Zin, 2009). While elastin fibers exhibit a linear stress-strain relationship up to 200% strain, they are important for elastic recoil and stabilization of lung parenchyma during normal tidal breathing (Yuan et al., 2000; Suki et al., 2011). In contrast, collagen fibers employ a highly non-linear stress-strain behavior as they become straight for larger strains and exhibit a high rigidity at larger strains. However, collagen and elastin are less expressed in lungs of preterm infants (Chrzanowski et al., 1980; Mižíková and Morty, 2015); together with a reduced amount of muscle fibers, the premature lung comprises poor elastic properties and is more susceptible to fatigue (Neumann and von Ungern-Sternberg, 2014). This low mechanical strength makes the lungs of infants highly sensitive to mechanical stress, e.g., when mechanical ventilation (MV) is necessary to ensure survival of diseased neonates. Technological advances such as high frequency oscillatory ventilation (HFOV) with small tidal volumes have promised to avoid overstretching of the lung, but have not eliminated ventilation-induced lung injury (Thome and Carlo, 2000; Thome, 2005; Cools et al., 2010), often resulting in bronchopulmonary dysplasia (BPD) (Gappa et al., 2006). Due to these side effects, MV constitutes a life-saving, but potentially injurious strategy for preterm infants, leading to BPD with a lifelong impairment of lung function in about half of the extremely low birthweight infants born in developed countries (Bell et al., 2022).

Besides altered ECM properties in preterm infants, dysfunction of alveolar cells also impairs the mechanical response of fetal lungs during inspiration. In fact, during fetal development, the lung is filled with fluid, which is actively secreted by lung epithelial cells to promote lung growth (Harding and Hooper, 1996). Prior to birth, the lung switches from fluid secretion to absorption to enable air breathing (Matalon and O’Brodovich, 1999). Mature alveolar type II (ATII) cells achieve this so-called alveolar fluid clearance (AFC) by unidirectional Na^+^ transport through epithelial Na^+^ channels (ENaC) and the Na,K-ATPase, creating an osmotic driving force for fluid absorption (Matalon and O’Brodovich, 1999). Impaired AFC due to insufficient expression of Na^+^ channels (O’Brodovich, 1996), and surfactant deficiency, as well as structural lung immaturity with low alveolar numbers, lead to fluid accumulation and alveolar instability. These effects compromise lung function and gas exchange, frequently resulting in BPD, the chronic lung disease of prematurity (Coalson, 2006). Furthermore, Na^+^ channels are mechanosensitive and can be expected to change function under mechanical load occurring during MV (Bogdan et al., 2008; Fronius and Clauss, 2008; Knoepp et al., 2020). Additionally, ATII cells secrete surfactant and an impaired cell function can therefore alter the alveolar surface tension and lung compliance during inspiration (Knudsen and Ochs, 2018).

To this end, understanding the mechanical properties of lung tissue and cells is urgently needed to elucidate the mechanisms that lead to ventilation-induced lung injury and subsequent BPD development. This would offer new opportunities to develop novel therapeutic approaches such as gentle MV modes to protect the vulnerable premature lung. In fact, even though alveolar cells and the entire lung are continuously deformed during inspiration and expiration, it is still not understood why inflation of the lung during MV, in contrast to normal tidal breathing with the same tidal volume, can cause respiratory damage–especially in preterm infants (Keszler and Durand, 2001; Hummler and Schulze, 2009; Neumann and von Ungern-Sternberg, 2014).

From a mechanical point of view, during normal tidal breathing and inspiration, a negative intrathoracic pressure results in a force, acting onto the outer lung walls (Palmer et al., 2004). Due to the created negative pressure in the lung compared to the outside, inspiration occurs and the lungs inflate. In contrast, during MV, a positive pressure inside the lung is created resulting in a force acting onto the inner lung walls and the lung expands. Since the lung as all living tissues is not fully elastic but a viscoelastic material (Eskandari et al., 2018; Birzle and Wall, 2019; Sattari and Eskandari, 2020; Corominas-Murtra and Petridou, 2021; Quiros et al., 2022), the hydrostatic pressure inside the lung tissue is expected to increase during MV compared to normal tidal breathing. If and to what extent changes in hydrostatic pressures affect lung tissue properties and cell behavior remains elusive.

In order to elucidate possible differences of lung tissue response during normal tidal breathing and MV, we studied fetal rat lungs and their mechanical properties under tension and compression using rheology experiments. Here we aim to understand tissue response during tension as happening during normal breathing, while in contrast the impact of tissue compression as occurring during mechanical ventilation was studied from a physics point of view. By applying different deformation velocities as present during lung inflation, we additionally correlated our results on lung mechanics on macroscopic length scale with the mechanosensitivity of cellular ion channels on microscopic scales. In fact, we investigated the vectorial Na^+^ transport in fetal distal lung epithelial (FDLE) cells, a model of preterm ATII cells, using an Ussing chamber to explore the influence of hydrostatic pressure in ranges occurring during normal tidal breathing.

## Materials and methods

### Lung tissue preparation

**FIGURE 1:**
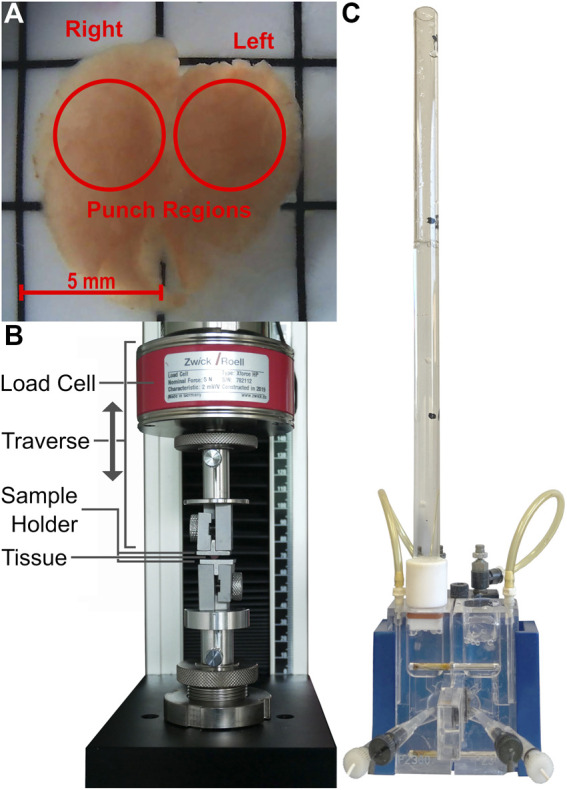
Images of the fetal rat lung after harvest **(A)**, the mechanical testing machine including load cell, as well as the self-designed sample holders **(B)** and the Ussing chamber with the applied fluid column **(C)**.

Lung tissue from fetal Sprague Dawley rats was used, while lung tissue of adult Sprague Dawley rats served as control. Rats were housed at the Medical Experimental Center (MEZ) of Leipzig University in standard conditions with a temperature of 22°C, 55% humidity and a 12 h-day-night-cycle. Food and water were available *ad libitum*. At 20–21 days post conception, pregnant rats were euthanized by pentobarbital-Na (150 mg kg^−1^) injection. This timing was chosen because fetal rats are in saccular stage of lung development, which relates to human preterm infants who often develop respiratory distress due to immature lung function. The abdomen was rinsed with 70%-alcohol, opened and rinsed again. Subsequently, the peritoneum was opened, and the uterus horns removed. Next, fetal rats and their heart-lung packets were obtained. Lungs were separated from the heart under a microscope and stored in Hank’s Balanced Salt Solution (HBSS, 14170138, Thermo Fisher Scientific, Germany) on ice. In addition, to eliminate surfactant of adult lungs, the sternum of selected euthanized adult rats was removed, and a tracheotomy was performed below the larynx. A cannula was inserted into the trachea and the lungs were initially rinsed with 1.5 ml of phosphate-buffered saline (PBS, 56064C, Sigma-Aldrich, Germany). This was followed by four additional rinses with 1 ml of PBS. Both the irrigated and the unirrigated lungs were removed and stored in HBSS. All experimental procedures were approved by the Leipzig University institutional review board (Landesdirektion Leipzig, T07/20) following the ethical guidelines on animal experiments. Overall, lungs of 54 pups and 18 adult rats were employed. Subsequently, rheology experiments were performed immediately. Here, the cranial parts of the left and right fetal lung lobes were punched perpendicular to the front plane with a 4 mm biopsy punch (BPP-40F, Kai Europe, Germany) (Figure 1A), while the lobes of the adult lung were punched with an 8 mm biopsy punch (15378, Jademed, Germany) to cover comparable areas of the lung. In case of the right lung, the punched area was therefore located in the superior lobe. The thickness of all fetal and adult samples (defined by the height of the punched cylinders) was measured as the distance between the sample plates of the mechanical test machine after gluing the samples to the plates (more details: see next section). We obtained values with standard errors of (2.0 ± 0.1) mm for the fetal samples and (4.2 ± 0.1) mm for the adult samples.

### Mechanical test arrangement

Quasi-static compressive and tensile tests were performed using the material testing machine 500 N zwicki (type Z0.5 TS, 058992, ZwickRoell GmbH & Co. KG, Ulm, Germany) equipped with a 5 N load cell (type Xforce HP, 063924, ZwickRoell GmbH & Co. KG, Ulm, Germany) and a data storage interval of 2 ms during experimental testing. The sample holders were self-constructed and consisted of two chambers in which interchangeable aluminum plates were clamped–see Figure 1B. Depending on the respective test, the plates were moved towards each other or apart with different velocities in a position-controlled manner. The upper sample holder was connected to the load cell to measure the force acting onto the sample, which was placed between the upper and lower sample holder. The testing machine was equipped with a DinoLite AM7915MZT (AnMo Electronics Corporation, Taipei, Taiwan) light microscope to check for possible sample slippage during experiments.

Prior to experiments, the upper sample holder was removed, whereupon the upper surface of the cylindrically punched sample was glued to it with ethyl 2-cyanoacrylate (Pattex® Sekundenkleber Classic flüssig–Mini Trio, 4015000415033, Henkel, Germany). The glue was used in compressive and tensile tests to avoid possible slippage and to ensure comparability of the data. A few seconds after gluing, the sample holder was connected to the test machine. Next, the glue was applied to the lower sample holder and both holders were moved towards each other so that the tissue sample touched the lower sample holder. Contact was reached when a contact force of approximately 1 mN was detected. During this procedure, the testing machine automatically determined the distance between the upper and lower sample holder, giving the height of the sample. In order to avoid drying of the sample, the tissue was kept moist by pipetting HBSS onto the sample.

After gluing and bringing the samples into contact with the sample holders, we waited for two to three minutes to allow the glue to harden. Subsequently, uniaxial compression and tension tests were performed with a maximum strain of 50%. Here the lower sample holder was fixed, while the upper holder (connected to the 5 N load cell) moved down towards the lower sample holder during compression or moved upwards to stretch the sample.

During each deformation, the force as function of distance was monitored. Additionally, a USB microscope (Dino-Lite Edge, AM7915MZT, AnMo Electronics Corporation, Taiwan) was installed next to the test machine to monitor tissue deformation during tension and compression and image possible slippage or detachment from the sample holders.

### Deformation velocities of lung tissue during tension and compression

All tension and compression experiments were performed at constant deformation velocities. This section described how we derived deformation velocities from clinical and other experimental data. In fact, in order to transfer breathing and MV frequencies used for preterm infants into deformation velocities employed for our experiments, we made the following assumptions: Very preterm infants often suffer from RDS, which can result in BPD (Ali et al., 2013; Thébaud et al., 2019). Considering that such neonates usually require MV to support breathing, an average tidal volume of 4–6 ml kg^−1^ and an average functional residual capacity (FRC) of 21.4 ml kg^−1^ can be assumed (Latzin et al., 2009; Chakkarapani et al., 2020). From the resulting volume increase of the lungs during inspiration, we obtained lung volume increases by about 18% $\left(\frac{4\frac{\mathrm{ml}}{\mathrm{kg}}}{\mathrm{21}\mathrm{.4}\frac{\mathrm{ml}}{\mathrm{kg}}}\cdot 100\approx 18\%\right)$ for gas supply of 4 ml kg^−1^ and 28% for 6 ml kg^−1^.

Approximating the lung as a homogeneous sphere, the calculated volume increase corresponds to a radius increase (linear strain during lung inflation) of 5.9–8.6%. Very preterm infants show an average inspiration time of 0.20–0.27 s (Brown and DiBlasi, 2011; Chakkarapani et al., 2020). To this end, for the calculated strains and cylindrically punched tissue samples of 2 mm lengths, a deformation velocity–*viz*. expansion velocity–in the range of 26 mm min^−1^ to 52 mm min^−1^ results.

However, as described by Vlahakis and Hubmayr (2005), a linear strain of the lung (*viz.* the expansion in diameter) and the corresponding cell stretch can reach values up to 15% without irreversible damage (*viz*. elastic deformation). Here, a maximum deformation velocity for an inspiration time of 0.27 s would be about 70 mm min^−1^ as used in our experiments.

In contrast, when considering an adult lung with an FRC of 30 ml kg^−1^ and a tidal volume of 6 ml kg^−1^ (Lipes et al., 2012), deformation velocities in the range of 3–7 mm min^−1^ are obtained for inspiration rates of 1–2 s (Sembroski et al., 2022).

Thus, in our experiments we employed a deformation velocity of 70 mm min^−1^ to mimic the maximum deformation velocity found in the preterm lung during inspiration, compared to 7 mm min^−1^ and 1 mm min^−1^ as examples of slower breathing frequencies, e.g., present in the adult lung and during conventional MV. The usage of alternative ventilation modes, such as HFOV (Fessler et al., 2007; Snoek et al., 2016), is also included at these velocities. Although higher frequencies of up to 600–800 breaths per minute are used here, the resulting lung expansion velocities are expected to be similar to the values calculated above because the tidal volumes are strongly reduced to reach the same final gas supply, which is approximately 0.2–0.3 L min^−1^·kg^−1^ in healthy neonates (Chakkarapani et al., 2020).

### Fetal distal lung epithelial cell isolation and culture

The protocol to isolate FDLE cells has been previously described in detail (Jassal et al., 1991; Thome et al., 2003). In brief, at 20–21 days post conception, fetal rat lungs were removed and mechanically dissociated. Subsequently, enzymatic digestion was performed by incubation in HBSS containing 0.125% trypsin (15090046, Thermo Fisher Scientific) and 0.4 mg ml^−1^ DNAse (LS006333, CellSystems, Germany) for 10 min at 37°C. Proteolytic activity was neutralized by the addition of Minimum Essential Medium (MEM, 21090055, Thermo Fisher Scientific) containing 10% Fetal Bovine Serum (FBS, S0615, Sigma-Aldrich). After various centrifugation steps, the cell suspension was filtered and resuspended in HBSS containing 0.1% collagenase (LS004196, CellSystems) and 0.4 mg ml^−1^ DNAse. Incubation was carried out at 37°C for 15 min, while the solution was vortexed intermittently. Digestion was stopped again by adding MEM with 10% FBS. After subsequent centrifugation and resuspension steps, cells were plated twice for 1 h each to remove fibroblasts. The resulting supernatant contained epithelial cells with a purity of more than 95% (Jassal et al., 1991).

For Ussing chamber experiments, cells were seeded at a density of 10^6^ cells per permeable Snapwell insert (3407, Corning, United States) with a surface area of 1.1 cm^2^ and a pore size of 0.4 µm. In case of a fluid column on the basolateral side of the Ussing chamber, Snapwell inserts were coated with 0.1 mg ml^−1^ human placental collagen type IV (C5533, Sigma-Aldrich) to ensure cell attachment during experiments. Here, a total of 612 FDLE monolayers were obtained from eight different cell isolations (153 fetuses from 16 pregnant rats). MEM containing 10% FBS, 2 mM L-glutamine (G7513, Sigma-Aldrich), 100 U·ml^−1^ penicillin (P0781, Sigma-Aldrich), 100 μg ml^−1^ streptomycin (P0781, Sigma-Aldrich) and 25 μg ml^−1^ amphotericin B (A4888, Sigma-Aldrich) was used to culture FDLE cells. Medium was changed daily.

### Ussing chamber measurements

Ussing chamber experiments were performed 72 h and 96 h after seeding FDLE cells. In order to investigate the effect of hydrostatic pressure differences on ion channel activity, we installed a fluid column-see Figure 1C-on the apical or basolateral side of the Ussing chamber, respectively. These could be filled with Ringer solution to a maximum height of 30 cm to maintain a constant pressure on one side of the cell monolayer during experiments. Only monolayers exceeding a transepithelial resistance (*R*_te_) of 300 Ω cm^2^ were included in the analysis (Geys et al., 2006). The Ringer solutions used in the chambers had a composition of: 145 mM Na^+^, 5 mM K^+^, 1.2 mM Ca^2+^, 1.2 mM Mg^2+^, 125 mM Cl^−^, 25 mM HCO_3_^−^, 3.3 mM H_2_PO_4_^−^ and 0.8 mM HPO_4_^2-^ with a pH of 7.4. To exclude possible influences of a potential apical Na^+^-glucose cotransporter (Icard and Saumon, 1999), 10 mM mannitol was used in the apical and 10 mM glucose in the basolateral solution. The solutions were heated to 37°C throughout the measurements and permanently aerated with a mixture of 5% CO_2_ and 95% O_2_.

A transepithelial current clamp (VCC MC8, Physiologic Instruments, United States) was used to measure the transepithelial potential difference (*V*_te_) and the transepithelial resistance (*R*_te_). Using Ohms’ law (*I*_sc_ = *V*_te_/*R*_te_), the equivalent short-circuit current (*I*_sc_) was calculated. The measured values were recorded every 20 s.

In case of an increased hydrostatic pressure on the basolateral side, we added 10 µM amiloride (A7410, Sigma-Aldrich) to the apical side after *I*_sc_ reached a stable plateau (*I*_base_). The amiloride-sensitive change in short-circuit current (∆*I*_sc_) was calculated to infer the activity of the ENaC under hydrostatic pressure. A maximum water column of 12.5 cmH_2_O was used on the basolateral side since larger pressures resulted in cell detachment. For determining the influence of hydrostatic pressures on the apical side, 1 mM ouabain (O3125, Sigma-Aldrich) was applied to the basolateral side after *I*_sc_ had reached a stable plateau. Herein, a maximum fluid column of 30 cmH_2_O was employed. The ouabain-sensitive ∆*I*_sc_ was determined as a measure of Na,K-ATPase activity under hydrostatic pressure. Addition of the respective inhibitor was only possible at chamber side not occupied by the hydrostatic pressure column, as this blocked access to the Ussing chamber. Amiloride and ouabain were dissolved in water.

Additionally, maximum amiloride-sensitive apical membrane permeability (*amil*_max_) was measured. This is used to determine the ENaC activity independent of the Na,K-ATPase. A 145:5 apical to basolateral Na^+^ gradient was obtained by replacing 140 mM basolateral Na^+^ with 116 mM N-methyl-D-glucamine (NMDG^+^, M2004, Sigma-Aldrich) and 24 mM choline (C7519, Sigma-Aldrich). *I*_sc_ was measured every 5 s with a transepithelial voltage clamp (VCC MC8, Physiologic Instruments). The basolateral membrane was permeabilized with 100 µM amphotericin B. The resulting *I*_sc_ arose only from the passive flow of Na^+^ through Na^+^ permeable pathways along the Na^+^ gradient from the apical to the basolateral side (Guo et al., 1998; Thome et al., 2001, 2003). When *I*_sc_ reached a maximum value, 10 µM amiloride was added basolaterally. Amiloride blocked the ENaC by passive flow to the apical side. Thereby, maximum ENaC activity was determined under hydrostatic pressure. A maximum fluid column of 30 cmH_2_O on the apical side was used. Amphotericin B was prepared in dimethyl sulfoxide (DMSO, D5879, Sigma-Aldrich).

In parallel, all experiments were conducted without the application of fluid columns–*viz*. no pressure difference between apical and basolateral compartment–as controls.

### Van der Waals model

In order to quantify the hyperelastic deformation behavior, we use the Kilian’s van der Waals model. In the van der Waals approach, a network of chains is assumed, with finite chain extensibility and global interactions within the network (Kraus et al., 1992). This model is phenomenological in nature and closely related to the van der Waals model of real gases being highlighted by the similarity of both equations of state (Kilian, 1982). Here, the equation of state of the van der Waals network is described by (Kraus et al., 1992):

$$f\left(\lambda \right)=G\cdot D\left(\lambda \right)\left(\frac{1}{1-\eta }-a\Phi^{\frac{1}{2}}\left(\lambda \right)\right)$$ (1)

with the strain *λ*, the nominal stress *f*(*λ*), the shear modulus *G*, the deformation function *D*(*λ*) = *λ–λ* ^−2^, *η* = (*Φ*/*Φ*_m_)^1/2^, *Φ*(*λ*) = ½ · (*λ* ^2^ + 2/*λ*—3) and *Φ*_m_ = *Φ*(*λ*_m_). Here, *a* and *λ*_m_ are the van der Waals parameters, where *a* characterizes global interactions across junctions, and *λ*_m_ is the maximum strain related to maximum chain extensibility (Kraus et al., 1992). Interactions of chains are considered as ‘collisions between quasiparticles’, where each chain behaves like a conformational gas particle (Kilian, 1981, 1989). Thus, on a global level, the van der Waals network exhibits the behavior of a conformational gas with weak interactions (Kilian, 1982). Consequently, a strain energy function can be defined (Kraus et al., 1992):

$$W\left(\lambda \right)=-G\left\{2\Phi_{m}\left[\ln\left(1-\eta \right)+\eta \right]+\frac{2}{3}a\Phi^{\frac{3}{2}}\right\}$$ (2)

Thereby, the relevance of this phenomenological model is the possibility to achieve a quantitative description in the entire strain domain of an experimentally determined stress-strain behavior by computing material properties of the underlying material network (Kilian and Vilgis, 1984).

Consequently, in order to obtain the lungs’ Young’s moduli not only by linear regression of the stress-strain behavior for small strains, we applied the hyperelastic van der Waals model. The analysis was performed for all measured stress-strain curves to determine the material parameters for larger deformation ranges using Abaqus Student Edition 2021 (Dassault Systémes Simulia Corporation, 2021). The model parameters *G*, *a* and *λ*_m_ appearing in Eq. 1 were determined using Abaqus in terms of the best fit. Assuming a Poisson’s ratio of *v* = 0.5 for biological tissue (Vito and Dixon, 2003; Wex et al., 2015), the shear modulus *G* was converted to the Young’s modulus *E* by:

E=2G(1+v) (3)

to better compare our data.

### Statistical analysis

For the rheology experiments, we performed 52 compression and 52 tension tests with fetal rat lungs at three different velocities described above, as well as 16 compression and 19 tension experiments with adult rat lungs at one velocity (7 mm min^−1^), totaling 139 deformation experiments (see Supplementary Table S1 for detailed information). The sample size of each group is approximately the same, resulting in no influence on the statistical analysis. From the resulting forces as function of distance, we calculated the strain (deformation distance relative to sample height) as well as the stresses (force per sample area). Subsequently, we performed linear regression of the linear regimes of the stress-strain plots for small strains (usually below 15%) using Origin 2019 and Python (McKinney, 2010; Rossum and Drake, 2010; Harris et al., 2020; Gazoni and Clark, 2021; Microsoft Corporation, 2021; OriginLab Corporation, 2021), as well as applying a hyperelastic van der Waals model using Abaqus over the entire strain range to determine the Young’s moduli (Dassault Systémes Simulia Corporation, 2021).

In case of electrophysiological experiments, we carried out 78 measurements, each with eight samples measured simultaneously. The three methods described above were used with up to four different pressures in addition to the control. Thus, each experiment was repeated 20–50 times.

Subsequently, mean values including standard errors were calculated for the respective experiments. Boxplots and bar charts were generated (Hunter, 2007; Rossum and Drake, 2010; OriginLab Corporation, 2021), and, for statistical analysis (Rossum and Drake, 2010; Vallat, 2018; Virtanen et al., 2020), the mean values of different data sets were compared. Here, a Shapiro-Wilk test for normal distribution was performed first, followed by a Bartlett’s test to check for variance homogeneity of the groups. Depending on these results, when comparing two groups, an independent Student’s t-test was performed, with Welch’s correction if necessary. When comparing several groups, an ANOVA followed by a Tukey’s post-hoc test was performed in case of variance homogeneity and a Welch’s ANOVA followed by a Games-Howell post-hoc test was performed when groups were heterogeneous in variance. Here a *p* value smaller than 0.05 was considered statistically significant. Statistical significance between groups is marked with * *p* < 0.05, ** *p* < 0.01, *** *p* < 0.001. Exact *p* values are provided in the Supplementary Material in Supplementary Table S2.

## Results

### Mechanical properties of fetal rat lungs

**FIGURE 2:**
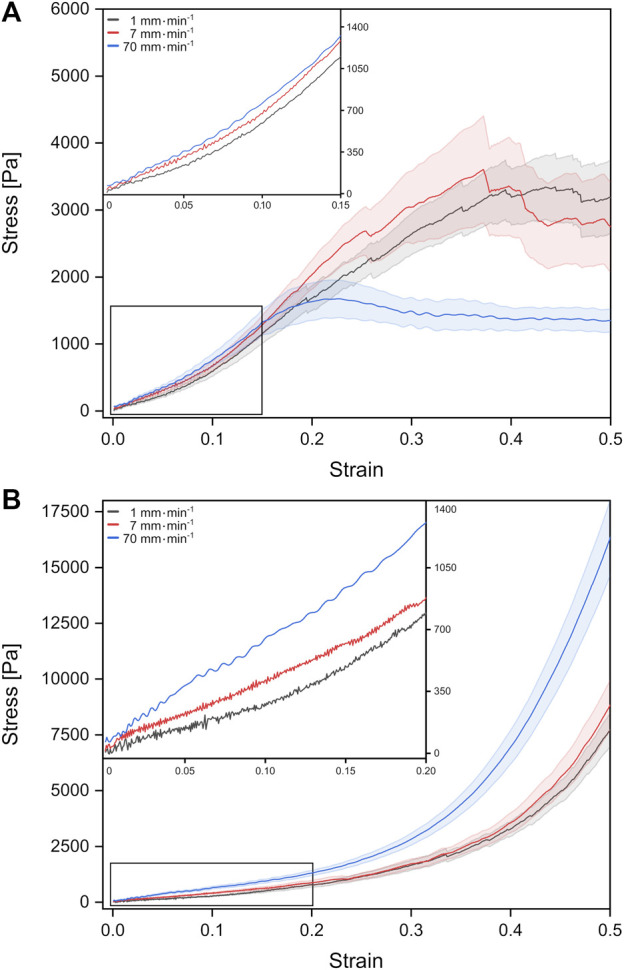
Stress-strain behavior of fetal rat lung tissue samples under tension **(A)** and compression **(B)**. The different curves represent mean values with the standard error obtained from individual tests with different probing velocities represented by colors (stress-strain data from individual test can be found in the Supplementary Figures S1, S2).

We investigated the mechanical properties of fetal rat lung tissue under tension and compression. From the resulting force-distance curves, we calculated the corresponding stress-strain behavior for three different velocities as shown in Figure 2. For all tension and compression experiments, we observed a linear stress-strain behavior for small strains below 15%, which bended over to non-linear behavior with an accelerated stress increase (individual stress-strain curves can be found in the Supplementary Material, Supplementary Figures S1, S2).

For fetal lung tissue under tension, a global stress maximum of about 1,674 Pa was reached fastest for the highest deformation velocity of 70 mm min^−1^ at a strain of around 22%. For velocities of 1 mm min^−1^ and 7 mm min^−1^, stress maxima were found at 3,347 Pa at 44% strain and 3,605 Pa for 37% strain, respectively.

Under compression, we observed no global stress maxima. Here the stress continuously increased with strain until the tissue compression was stopped at 50% strain. In summary, faster velocities resulted in larger stresses at 50% strain with 16,314 Pa for 70 mm min^−1^ compared to 7,691 Pa for 1 mm min^−1^. We would like to point out that local stress maxima with amplitudes of the order of 100–200 Pa occurred in some compression experiments resulting from possible local mechanical failure and release of fluids (Supplementary Figure S2).

### Determination of Young’s moduli

**FIGURE 3:**
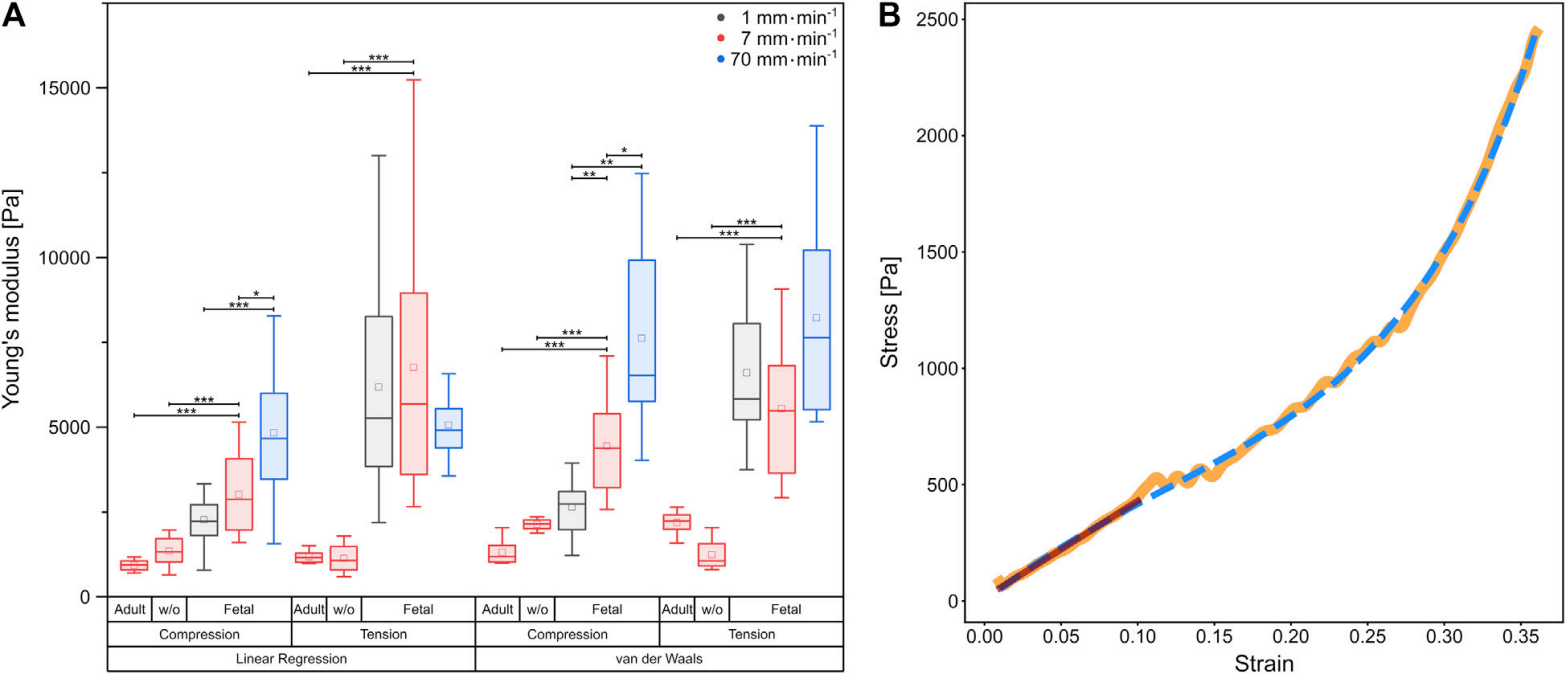
**(A)** Boxplot of Young’s moduli determined by linear regression, as well as calculation of the van der Waals model, respectively. Underlying data were obtained from compression and tension tests of fetal and rinsed (w/o) and unrinsed adult rat lung samples with different velocities represented by colors. Circles show mean values, while central lines represent medians (50th percentile); the bottom and the top of the box (hinges) represent 1st and 3rd quartiles; the end of the whiskers represent the minimum and maximum of the data (hinges ±1.5 interquartile range). **(B)** Comparison of linear regression (red) with the van der Waals model (blue dashed) on experimental data (orange) from a compression test at 7 mm⋅min^−1^ using a fetal rat lung.

In order to compare the elastic behavior of the tissue samples for small strains in tension and compression, we determined the slope of the stress-strain curves within the linear regime below 15% strain. The resulting Young’s moduli are summarized in Figure 3A.

For all compression experiments with fetal rat lung samples, the Young’s moduli obtained by linear regression increased significantly with deformation velocity, while for tensile experiments, the Young’s moduli did not differ significantly. In general, we determined smaller Young’s moduli under compression compared to tension [v = 1 mm min^1^ (2,270 ± 211) Pa under compression and (6,183 ± 767) Pa under tension, v = 7 mm min^−1^ (3,011 ± 304) Pa under compression and (6,767 ± 1,091) Pa under tension], while for the fastest probing velocity of 70 mm min^−1^, similar values were found [compression (4,829 ± 514) Pa, tension (5,059 ± 306) Pa]. Thus, while for compression a strong velocity dependence of mechanical properties became evident, the elastic moduli remained almost constant under tension. However, the stress-strain behavior in Figure 2 with the overbending–*viz*. highly non-linear–stress increase following the linear regime for small strains points towards a hyperelastic material property characteristic for rubber networks. Thus, considering the lung as a network composed of collagen and elastin (Suki et al., 2005), we verified the measured material properties of the fetal lungs over the entire deformation range by employing the phenomenological Kilian’s van der Waals network model (see Materials and methods) as shown in Figure 3B. The determined material parameters are shown in Table 1 and the Young’s moduli in Figure 3A.

**TABLE 1:** Mean parameters with standard error derived from the van der Waals model of the best fits to the experimental data for the respective lung samples at different velocities. Here, *E* is the Young’s modulus, λ_m_ is the maximum strain, and α is the parameter describing the global interactions within the network. Adult (rinsed) refers to the adult lung without surfactant.

Age	Test	Velocity [mm·min^−1^]	*E* [Pa]	*λ*_m_	*a*
Fetal	Compression	1	2,646 ± 263	2.1 ± 0.1	1.9 ± 0.2
		7	4,441 ± 415	2.10 ± 0.04	3.0 ± 0.2
		70	7,617 ± 1,059	2.12 ± 0.04	2.7 ± 0.5
	Tension	1	6,607 ± 696	1.9 ± 0.1	5.8 ± 1.6
		7	5,545 ± 600	1.86 ± 0.02	3.2 ± 0.9
		70	8,221 ± 1,081	1.81 ± 0.02	4.3 ± 0.9
Adult	Compression	7	1,311 ± 130	2.2 ± 0.1	2.7 ± 0.5
	Tension	7	2,187 ± 118	1.9 ± 0.03	4.3 ± 0.7
Adult (rinsed)	Compression	7	2,135 ± 231	2.7 ± 0.2	1.7 ± 0.4
	Tension	7	1,239 ± 273	1.8 ± 0.03	4.3 ± 1.3

Calculation from the van der Waals model obtained for tissue under compression showed a significant rise in Young’s moduli with increasing probing velocities [v = 1 mm min^−1^ (2,646 ± 263) Pa, v = 7 mm min^−1^ (4,441 ± 415) Pa, v = 70 mm min^−1^ (7,617 ± 1,059) Pa]. In contrast, under tension hardly any significant differences became evident as shown in Figure 3A. However, here the values were significantly larger than those under compression [v = 1 mm min^−1^ (6,607 ± 696) Pa, v = 7 mm min^−1^ (5,545 ± 600) Pa, v = 70 mm min^−1^ (8,221 ± 1,081) Pa]. In summary, the material properties under compression and tension differed greatly: fetal rat lungs can be assumed to be softer under compression with lower Young’s moduli than under tension. Additionally, the tissue’s elasticity was deformation velocity independent under tension but highly velocity-dependent under compression.

### Mechanical properties of adult rat lungs and impact of surfactant

**FIGURE 4:**
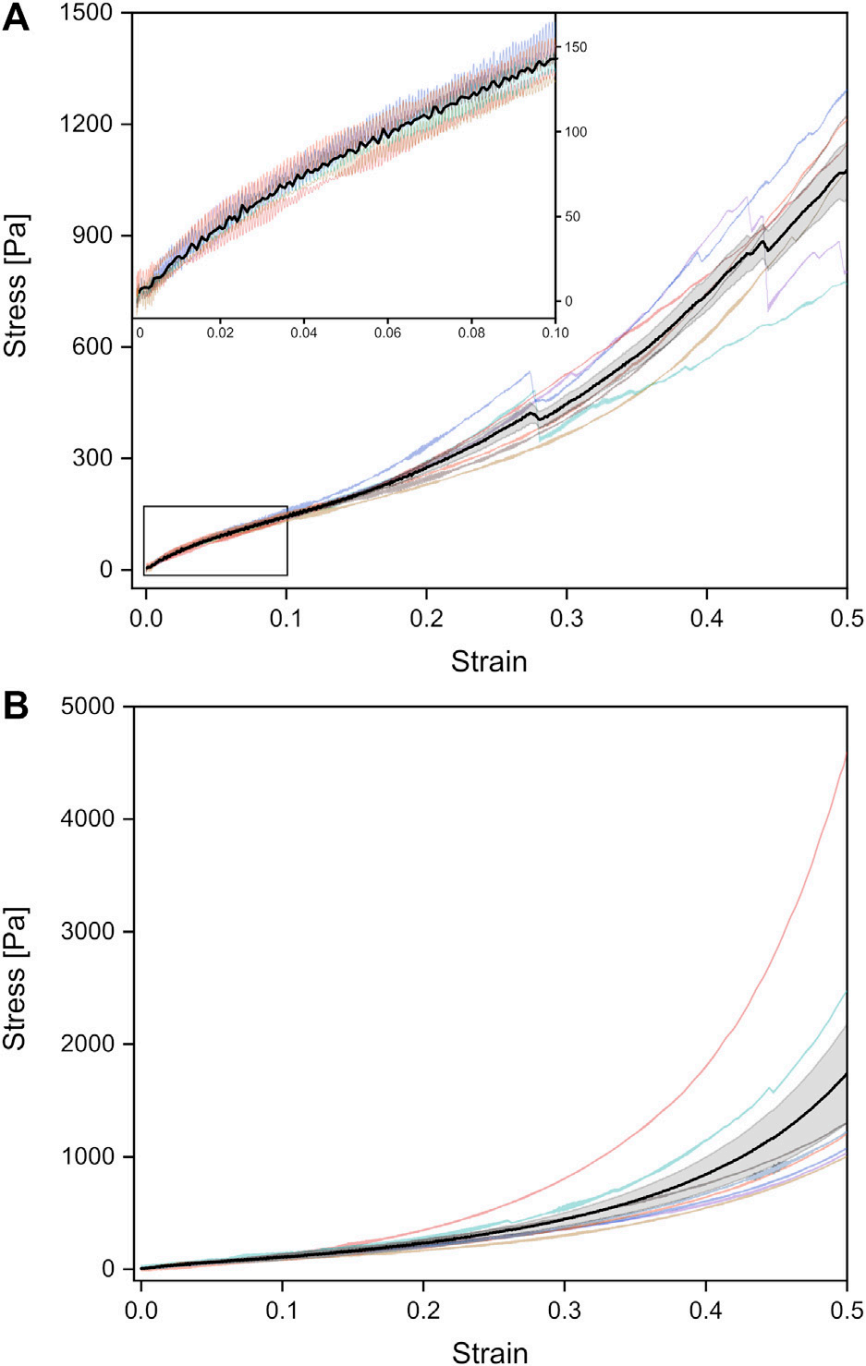
Stress-strain curves of unrinsed (*viz.* surfactant-containing) adult rat lungs tissue samples under tension **(A)** and compression **(B)**, obtained from a probing velocity of 7 mm⋅min^−1^. The black curves represent the mean values with the standard error obtained from all individual tests shown in different colors.

Furthermore, we determined the deformation behavior of adult rat lung samples obtained from mother rats at a deformation velocity of 7 mm min^−1^. The adult tissues showed a similar stress-strain behavior under compression compared to the fetal lungs with a continuous stress increase, while the overall stress values were smaller with maximum stresses below 1,741 Pa for 50% strain (Figure 4B). For small strains during compression, an average Young’s modulus of (932 ± 59) Pa was found, while for the van der Waals model, a value of (1,311 ± 130) Pa was obtained, both significantly smaller than their fetal counterpart.

Under tension, the Young’s modulus of linear regression was slightly larger with (1,178 ± 64) Pa compared to compression. A larger discrepancy occurred in the van der Waals model with a Young’s modulus of (2,187 ± 118) Pa under tension. As shown in Figure 3A, significant differences between compression and tension appeared both in linear regression and van der Waals model (Supplementary Figure S3). Striking was the different stress-strain behavior under tension: From seven individual experiments, three tissue samples showed local stress maxima because of rupture during tension, while in four experiments the stress continuously increased until 50% strain (end of tissue expansion) (Figure 4A). The maximum stresses for 50% strain were of the order of 1,100 Pa for tissue under tension and around 1,700 Pa for compression–about 3–4 times smaller compared to values found for fetal lungs.

We would like to point out that besides the different Young’s moduli of the adult lungs compared to the fetal counterparts, also the shape of the stress-strain curves varied. Fetal lungs exhibited a highly hyperelastic behavior under tension and compression with accelerating stress values for increasing strain. In contrast, adult tissues showed a “classical” deformation response with a linear strain until about 5% and a slowed-down stress increase succeeding the linear regime until about 15% strain.

In addition, we measured rinsed lungs of adult rats in identical rheology experiments in order to investigate a possible impact of surfactant on lung mechanical response. For surfactant-depleted adult lungs, values of (1,346 ± 159) Pa under compression and (1,134 ± 149) Pa under tension were found when linear regression was used. No significant differences were seen for rinsed and unrinsed lungs and Young’s moduli of adult lungs were always smaller compared to fetal lungs (Figure 3A). When we employed the van der Waals model which considered stresses in a much larger stress regime compared to linear regression, we obtained an increase in Young’s modulus without surfactant in tension mode and a decrease in Young’s modulus during compression. However, due to the small number of available adult rats, we do not consider these variations in data analysis as statistical significant and our results clearly show that a lack of surfactant is not the origin of the mechanical differences between adult and fetal lungs.

### Ussing chamber measurements

Besides the macroscopic deformation behavior due to acting external load, we determined the impact of acting forces in terms of hydrostatic pressure at the cellular level by performing Ussing chamber experiments with FDLE cell monolayers. By including a fluid column on one side of the Ussing chamber, we increased the hydrostatic pressure acting on the basolateral or apical side, respectively. Here hydrostatic pressure-related differences in vectorial Na^+^ transport were investigated, with chosen pressures up to 30 cmH_2_O based on pressure differences present during tidal breathing.

By measuring transepithelial potential *V*_te_ and short-circuit current *I*_sc_, as well as investigating amiloride-sensitive apical membrane permeability *amil*_max_; we aimed to determine the influence of hydrostatic pressure on ENaC and Na,K-ATPase.

**FIGURE 5:**
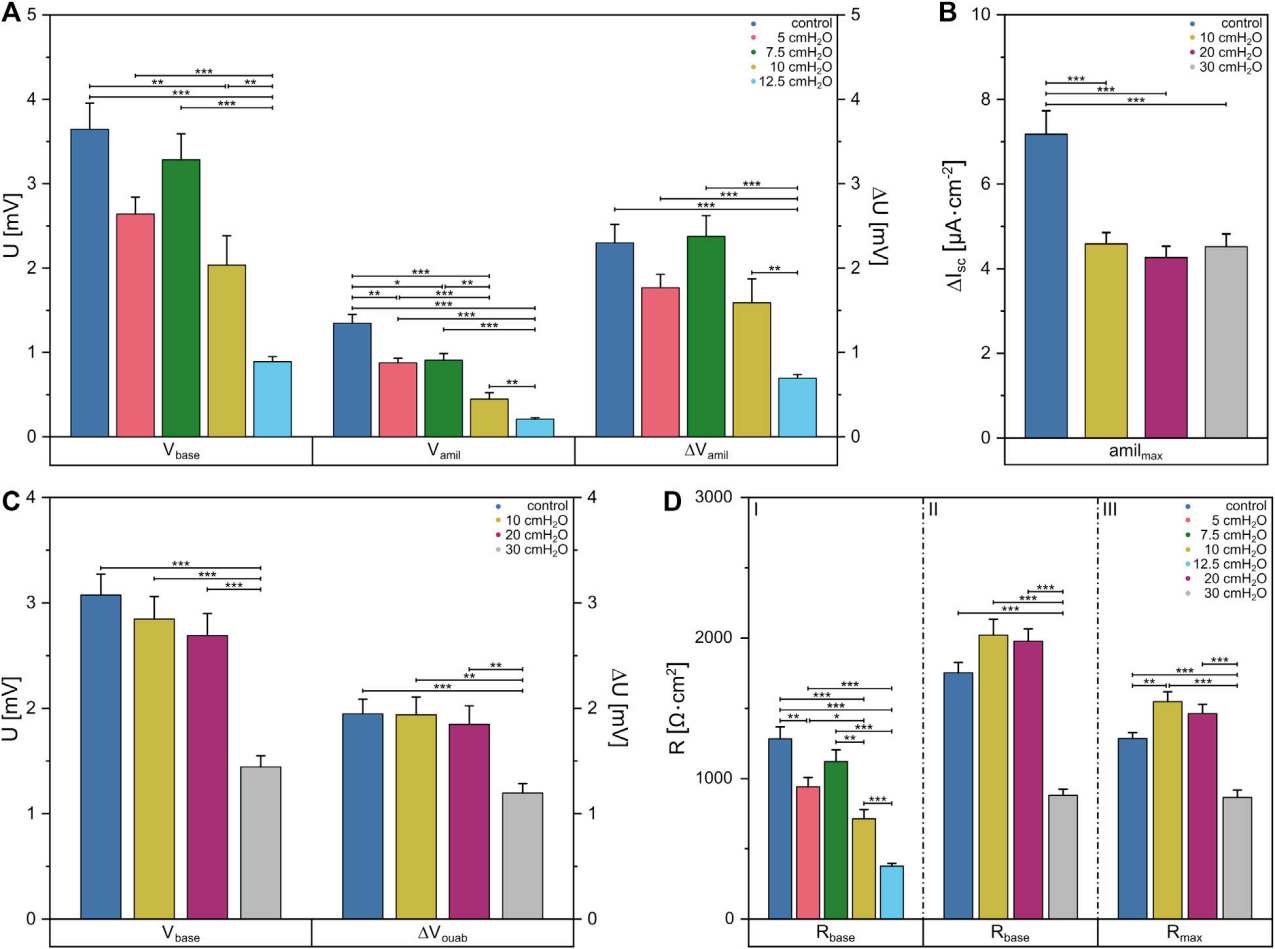
Data obtained from Ussing chamber experiments in which the apical or basolateral side of FDLE cells was suspended to an increased hydrostatic pressure. Column diagram of **(A)** *V*_te_ under basal conditions (*V*_base_), amiloride-insensitive *V*_te_ (*V*_amil_) and the amiloride-sensitive *V*_te_ (Δ*V*_amil_) with basolateral fluid columns, **(B)** the amiloride-sensitive component of the amphotericin-induced maximal *I*_sc_ (*amil*_max_) with apical fluid columns and **(C)** *V*_te_ under basal conditions (*V*_base_) and the ouabain-sensitive *V*_te_ (Δ*V*_ouab_) with apical fluid columns. **(D)** Column diagram of *R*_te_ after *I*_sc_ reached a stable plateau in amiloride (I) and ouabain (II) measurements or the maximum value in *amil*_max_ (III) measurements, respectively. The columns represent the mean values emerging from individual measurements with application of different fluid columns represented by colors.

To this end, we first determined the amiloride-sensitive *V*_te_ (Δ*V*_amil_) of FDLE monolayers for basolateral fluid columns up to 12.5 cmH_2_O since larger columns led to cell detachment. Here, mean *R*_te_ decreased with increasing pressures [*p* = 0 cmH_2_O (1,283 ± 84) Ω·cm^2^, *p* = 12.5 cmH_2_O (377 ± 20) Ω·cm^2^] (Figure 5D I). Further, significant differences in *V*_base_ were observed between the control group with (3.6 ± 0.3) mV and 10 cmH_2_O with (2.0 ± 0.3) mV, as shown in Figure 5A. Subsequent increases in basolateral hydrostatic pressure led to a decrease of *V*_base_, whereby it was lowest at 12.5 cmH_2_O with (0.9 ± 0.1) mV and showed significant differences to all other groups [*p* = 5 cmH_2_O (2.6 ± 0.2) mV, *p* = 7.5 cmH_2_O (3.3 ± 0.3) mV]. The amiloride-sensitive *V*_te_ (Δ*V*_amil_), likely related to Na^+^ transport, behaved similarly [*p* = 0 cmH_2_O (2.3 ± 0.2) mV, *p* = 5 cmH_2_O (1.8 ± 0.2) mV, *p* = 7.5 cmH_2_O (2.4 ± 0.2) mV, *p* = 10 cmH_2_O (1.6 ± 0.3) mV, *p* = 12.5 cmH_2_O (0.69 ± 0.04) mV]. However, amiloride-insensitive *V*_te_ (*V*_amil_), likely related to Cl^−^ transport, showed differences when comparing pressures of 5 cmH_2_O with (0.9 ± 0.1) mV to the control group with (1.3 ± 0.1) mV, while a significant decrease of *V*_amil_ occurred with increasing pressures up to 12.5 cmH_2_O [*p* = 7.5 cmH_2_O (0.9 ± 0.1) mV, *p* = 10 cmH_2_O (0.4 ± 0.1) mV, *p* = 12.5 cmH_2_O (0.21 ± 0.02) mV].

By applying increased hydrostatic pressure on the apical side, no differences in mean *R*_te_ between the control group with (1,752 ± 72) Ω·cm^2^ and higher pressures of 10 cmH_2_O and 20 cmH_2_O were found. One could only see a decrease in *R*_te_ to (879 ± 44) Ω·cm^2^ compared to the other groups when using a fluid column of 30 cmH_2_O (Figure 5D II). The same behavior was seen for *V*_base_ (Figure 5C), in which no differences were found between the control group and the lower pressures [*p* = 0 cmH_2_O (3.1 ± 0.2) mV, *p* = 10 cmH_2_O (2.8 ± 0.2) mV, *p* = 20 cmH_2_O (2.7 ± 0.2) mV], but at 30 cmH_2_O *V*_base_ was significantly lower with a value of (1.4 ± 0.1) mV. This was also evident in the measurements of the ouabain-sensitive *V*_te_ (Δ*V*_ouab_) [*p* = 0 cmH_2_O (1.9 ± 0.1) mV, *p* = 10 cmH_2_O (1.9 ± 0.2) mV, *p* = 20 cmH_2_O (1.8 ± 0.2) mV, *p* = 30 cmH_2_O (1.2 ± 0.1) mV].

Using fluid columns with respective hydrostatic pressures on the apical side, we determined the maximum Na^+^ permeability (*amil*_max_) independent of the Na,K-ATPase. The mean *R*_te_ showed a significantly lower value only in 30 cmH_2_O with (863 ± 53) Ω·cm^2^ compared to the other groups [*p* = 0 cmH_2_O (1,282 ± 43) Ω·cm^2^, *p* = 10 cmH_2_O (1,546 ± 69) Ω·cm^2^, *p* = 20 cmH_2_O (1,461 ± 65) Ω·cm^2^] (Figure 5D III). It was found that *amil*_max_ was significantly lower at each pressure [*p* = 10 cmH_2_O (4.6 ± 0.3) μA·cm^−2^, *p* = 20 cmH_2_O (4.3 ± 0.2) μA·cm^−2^, *p* = 30 cmH_2_O (4.5 ± 0.3) μA·cm^−2^] compared to the control group with (7.2 ± 0.3) μA·cm^−2^ (Figure 5B). *V*_te_ dropped significantly in apical measurements with ouabain at a fluid column of 30 cmH_2_O, whereas in apical *amil*_max_ measurements the *I*_sc_ dropped significantly at a height of 10 cmH_2_O indicating altered cell function under hydrostatic pressure.

## Discussion

Besides the heart, lungs are under constant periodical stress and deformation (Lai-Fook, 1977; Knudsen and Ochs, 2018). Especially in very early neonates when the lung is not fully developed (Merkus et al., 1996), many infants suffer from RDS characterized by a lowered lung compliance and a reduced lung volume (Yadav et al., 2022). Even though MV can be a life-saving strategy, the risk of ventilation-induced lung injury and development of BPD must be considered (Ali et al., 2013; Thébaud et al., 2019; Kalikkot Thekkeveedu et al., 2022; Yadav et al., 2022). It is still not fully understood why inflation of the lung during MV can result in irreversible damage in contrast to an increase in lung volume during normal inspiration. To elucidate the impact of mechanical load–tension as acting force during normal inspiration vs. tissue compression occurring during MV–we investigated the mechanical response of fetal and adult rat lung tissue during uniaxial rheology experiments. Here different deformation velocities relating to the inhalation speed of tidal breathing and MV frequencies were employed to determine the lungs’ Young’s moduli on a macroscopic length scale.

For small strains up to approximately 15% under compression and 10% under tension, we observed a linear elastic response of fetal rat lungs for all deformation velocities, which is in the physiologically relevant range of lung tissue deformation during respiration (Vlahakis and Hubmayr, 2005). However, for larger strains of the fetal lung samples, highly nonlinear stress-strain behavior was observed, characterized by an accelerating stress increase, a feature of hyperelasticity (Birzle et al., 2019b). Additionally, for the tension experiments, we observed that a maximum stress plateau was reached fastest and with smallest maximum global stresses compared to tension experiments with slower deformation velocities. We made similar observations for adult mammalian retinae under tension. Such effects are attributed to the given time scales of internal relaxation processes (*viz.* ergodic contributions due to thermal relaxation or non-ergodic effects occurring during local bond rupture) which are affected by the time scales of the external deformation (Juncheed et al., 2020).

Moreover, we observed strain stiffening of fetal lung tissue during compression, as well as tension. In contrast, adult tissues showed a different response as similarly found for adult lung tissues reported by van Oosten et al. (2019) and Andrikakou et al. (2016): for strains up to about 15%, strain stiffening is only seen under compression. Under tension softening occurred with a slowed-down stress increase following the linear elastic regime (Figure 4A, top left inset). Nevertheless, also for the adult lungs, Young’s moduli were always smaller under compression compared to tension. As described by van Oosten et al. (2019), tissues stiffen in compression and remain constant or even get softer under tension. In contrast, crosslinked biopolymers such as collagen soften in compression and stiffen under tension (van Oosten et al., 2019). Thus, in our experiments fetal lung tissues represent deformation features which combine the mechanical response of tissues and semiflexible biopolymers and therefore behave differently than adult lungs described before (Andrikakou et al., 2016; van Oosten et al., 2019). We expect that the mechanical properties of fetal lungs are determined by the complex lung structure of millions of alveoli in combination with the pulmonary elastin-collagen ECM network of the lung architecture (Suki et al., 2005; Knudsen and Ochs, 2018; Birzle et al., 2019a). Stiffening of the lung under tension might be due to the limited stretch behavior of the collagen fibers (Maksym and Bates, 1997; Suki et al., 2005; Faffe and Zin, 2009), while during compression, stiffening is more likely due to internal friction effects, interactions between filaments and cells, or fluctuations of the filaments (van Oosten et al., 2019).

As reviewed by Knudsen and Ochs (2018), the lung deformation is mainly determined by surfactant for small strains; the mechanical response for larger strains depends on the ECM properties composed of elastin and collagen (Suki et al., 2005; Knudsen and Ochs, 2018; Birzle et al., 2019a). In fact, fetal rat lungs had not yet formed surfactant in their developmental stage related to neonates (Merkus et al., 1996), and to exclude the influence of surfactant on the mechanical properties, we examined rinsed adult rat lungs and compared those with the fetal samples. Nevertheless, no differences were found between rinsed and unrinsed adult rat lungs, which could explain the much smaller Young’s moduli of adult lungs compared to fetal lungs.

Linking compression and tension to lung physiology and ventilation treatments, our tensile tests mimic normal tidal breathing, in which expansion of the lungs was caused by a negative intrathoracic pressure resulting in a force pulling the outer lung surface towards the chest walls (Palmer et al., 2004). The compression tests can thereby be associated with MV, since expansion of the lungs was achieved by creating an overpressure. Here the hydrostatic pressure increased and the tissue compressed since it is not fully elastic. The observed strain stiffening might be considered as a protecting mechanism to avoid overstretching for larger strains. Considering that fetal lungs behaved softer for small strains around 15% under compression with a smaller Young’s modulus compared to tension, we propose that fetal lung tissues might easier overstretch during inflation caused by MV compared to lung expansion due to negative intrathoracic pressure.

In order to reduce the ventilation volume for young neonates to avoid the risk of lung injury, various MV-techniques such as HFOV with 300–1,200 breaths per minute can be employed (Thome and Carlo, 2000; Fessler et al., 2007; Snoek et al., 2016), several orders of magnitude faster than the normal respiratory rate of 30–60 breaths per minute in healthy newborns (Reuter et al., 2014). In this study, we used different deformation rates corresponding to different breathing and ventilation frequencies to determine the impact of stretch and compression velocity on fetal lung tissue response. We found that for physiologic and ventilatory velocities, the Young’s modulus was unchanged during tension–a clear indicator of fully elastic deformation for small strains. In contrast, fetal lungs under compression became stiffer with increasing velocity which gives rise to viscoelastic properties of the tissue under the applied loading conditions (Ferry, 1980). Here, even in the elastic deformation regime for small strains, local rupture events such as bond breaking and irreversible molecular reorganizations and friction between tissue component can result in non-affine, *viz.* plastic deformation at the microscale (Juncheed et al., 2020; Zapp et al., 2020). We would like to point out that a change of fluid motion within the poroelastic network of the lung might also contribute to the observed deformation speed dependency. Since we already observed a strong deformation rate dependency for other densely packed tissues such as the retina under uniaxial deformation (Juncheed et al., 2020), we expect that the contribution of porosity to the rate-dependency is small and viscoelasticity is the key. Nevertheless, poroelasticity calculations (Berger et al., 2016, 2017; Concha and Hurtado, 2020) might be an interesting tool to distinguish between the contributions of the porous network structure of the lung including fluid flux and the material properties such as ECM network-cell interaction to the entire lung mechanical response. Relating the observed rate-dependence of compression to MV, we expect an increased lung compliance for higher frequencies since the fetal lungs stiffened with deformation velocity. From a materials science point of view, viscoelastic properties as seen from the rate-dependent mechanical response became more dominant for small strains under compression compared to tension. Thus, we expect that ventilation-induced injury of the diseased preterm lung can already happen when small ventilation volumes are employed and the protecting chest wall present *in vivo* cannot prevent the lung from overstretching.

The observed mechanical features of lung tissues are expected to be dominated by the high elastin content of the lung (Chrzanowski et al., 1980; Mižíková and Morty, 2015), since it is known that, e.g. the hyperelastic behavior of aortic tissues is mainly caused by the high elastic content (Rosenbloom et al., 1993). In fact, elastin can be assumed to play a major role in lung expansion during inspiration, whereas collagen limits its stretch behavior (Maksym and Bates, 1997; Suki et al., 2005; Faffe and Zin, 2009; Birzle et al., 2019a). Altered elastin content, as well as crosslinking differences of the lungs in preterm infants compared to adults may serve as a key role in the observed mechanical differences (Chrzanowski et al., 1980; Mižíková and Morty, 2015). Moreover, as shown by Wilharm et al. (2019), buckling instabilities such as Euler buckling in elastin networks, can enforce structural failure (Han et al., 2013; Nelson, 2016), and the network collapses irreversibly under a certain load. Such behavior is not seen in our experiments since the tissue is most likely stabilized by incompressible cells, which, in case of tissue compression, impede a network collapse. However, the impact of elastin as a hyperelastic material (Zou and Zhang, 2009) is seen when Young’s moduli were calculated from linear regression of small strains compared with computed data using a Kilian’s van der Waals model for the entire strain range (Kilian and Vilgis, 1984). While Young’s moduli from both calculations were in the same order of magnitude, the van der Waals potential also uses the large strains for the Young’s modulus calculation and therefore takes the hyperelastic features into account. Thus, the van der Waals calculations result in slightly increased moduli. Van der Waals potential parameters such as the maximum strain *λ*_m_ or the parameter *a*, which is significant for the global interactions in the network (Kraus et al., 1992), were also determined. While *λ*_m_ was constant for the different data sets, also the network interaction parameter *a* was always of the same order of magnitude. These observation supports our applied Kilian model as suitable fit to calculate the lungs’ tissue modulus, even though in the hyperelastic model the material’s Young’s moduli are considered to be deformation rate independent (Bower, 2016).

The effect of positive pressure and hydrostatic pressure gradients on cellular function was approached by investigating the vectorial Na^+^ transport *via* the ENaC and the Na,K-ATPase by electrophysiological experiments with an Ussing chamber. Here pressure gradients in the physiologic range up to 30 cmH_2_O were applied, and we measured *V*_te_ and *R*_te_ across polarized FDLE cell monolayers. A reduction of Na^+^ transport was observed at pressures as low as 10 cmH_2_O in case of basolateral positive pressure and when applying a hydrostatic pressure on the apical side at pressures of 30 cmH_2_O. During apical fluid column application, the ENaC is not accessible to its inhibitor amiloride. We thus permeabilized the basolateral membrane with an apically applied fluid column to obtain *amil*_max_ measurements, which reflect the maximal ENaC activity under elevated pressure. Under these circumstances, a pressure of 10 cmH_2_O was already sufficient to reduce ENaC-mediated Na^+^ transport. Prior studies demonstrated that preterm infants with RDS had reduced epithelial Na^+^ transport (Barker et al., 1997) and reduced ENaC expression (Helve et al., 2004) compared with preterm infants without RDS or term infants. Moreover, decreased AFC has been shown to contribute to the pathogenesis of RDS (O’Brodovich, 1996). With regard to these studies, a reduction of Na^+^ transport during positive pressure as observed in our experiments could further aggravate the critical situation for vulnerable preterm infants and worsen their clinical outcome. Moreover, hydrostatic pressure affected epithelial integrity as shown by the significantly reduced *R*_te_ in Ussing chamber experiments. The alveolar barrier prevents the exudation of fluid from capillaries into the alveolar spaces and contributes to the fluid absorption from the alveolar spaces. Disruption of the epithelial integrity may result in deleterious consequences, antagonizing AFC and leading to alveolar flooding (Yanagi et al., 2015), which is detrimental for preterm lung function.

Mechanosensitivity of the EnaC in endothelial cells was recently demonstrated by Knoepp et al. (2020), who proposed that shear forces can activate these ion channels in order to regulate blood pressure (Fronius and Clauss, 2008). In the lung, ENaC is responsible for the AFC (Garty and Palmer, 1997; de la Rosa et al., 2000). Thus, positive pressures, even orders of magnitude smaller than maximum pressures used during HFOV (Fessler et al., 2007; Snoek et al., 2016) might significantly impair ion channel activity and therefore affect fluid regulation in the preterm lung. In fact, mechanical force can produce a net inhibition of *I*_sc_, which consequently leads to a net decrease in ion absorption (Bogdan et al., 2008). Additionally, one has to keep in mind that fluid-driven deflection and fluid motion within the poroelastic lung tissue might also affect tissue elasticity and ion channel activity (Berger et al., 2016).

Considering the observed alterations in mechanical properties of fetal lung tissue under compression together with the impact of hydrostatic pressure on ENaC activity in fetal distal lung epithelia, we expect that ventilation techniques must carefully address correlations between tidal volumes, pressures and frequencies. As demonstrated by Kaczka et al. (2015) and Herrmann et al. (2020), in contrast to HFOV and conventional MV, multi-frequency oscillatory ventilation modalities (MFOV) might provide improved lung-protective ventilation by reducing strain magnitudes and spatial gradients of strain.

## Conclusion

During a lifespan, the lung periodically expands for up to 10^9^ cycles, as calculated by Fredberg and Kamm (2006). Thus, they concluded: “By the standards of common engineering materials, these strains are extreme and would appear to call for tissue structures that are rather substantial”. From a material’s science perspective, tissues can be considered as viscoelastic materials, which deformation behaviors depend on deformation rate and loading range. In our experiments, we observed that fetal rat lung tissues under tension behave fully elastically up to about 10–15% with no deformation rate dependency when velocities comparable to inspiration or MV rates are used. In contrast, during compression, even for small strains in the elastic regime, viscoelastic properties characterized by a high deformation-rate dependency become prominent, indicating possible non-affine, *viz.* irreversible events such as rupture on molecular level (Juncheed et al., 2020; Zapp et al., 2020).

Since tissue compression and an increased hydrostatic pressure in the lung are highest during HFOV with ventilation pressure amplitudes up to 100 mbar (Fessler et al., 2007), especially lungs of early neonates might be exposed to an increased risk of damage and ventilation-induced injury. Together with our findings that an increased positive pressure hinders alveolar epithelial Na^+^ transport, we conclude that MV even with small tidal volumes not only increases the risk of cell damage due to oxidative stress but can also cause molecular alterations and mechanically induced dysfunction of alveolar epithelial cells important for surfactant secretion and AFC. Thus, in order to avoid ventilation-induced injury, the development of new strategies for safe respiratory support in preterm infants are demanding (Berger et al., 2013). Following Stocks et al.’s (2013) statement that only better knowledge of the developing lung during prenatal and postnatal life can help to decrease the rising respiratory morbidity throughout life, this study might contribute to improved ventilation strategies which take mechanical differences of premature lungs in contrast to adult lungs into account.

Moreover, ventilation-induced lung injury has also the potential to cause morbidity and mortality of adult patients. Thus, our finding might also be adaptable to the adult lung (Ak and Anjum, 2022) and could give rise to a better understanding of treatment-related complications of patients suffering from COVID-19 pneumonia (Cronin et al., 2021) and other lung diseases. Future studies will focus on structural differences of fetal vs. adult ECM components and how their interplay alters tissue mechanics under varying loading conditions.

## Data Availability

The original contributions presented in the study are included in the article/Supplementary Material, further inquiries can be directed to the corresponding author.
