# Supplementary figures and images for: Mechanical properties of the premature lung: From tissue deformation under load to mechanosensitivity of alveolar cells
Source: Front Bioeng Biotechnol. 2022 Sep 16;10:964318. doi: 10.3389/fbioe.2022.964318 (PMC9523442; doi:10.3389/fbioe.2022.964318)

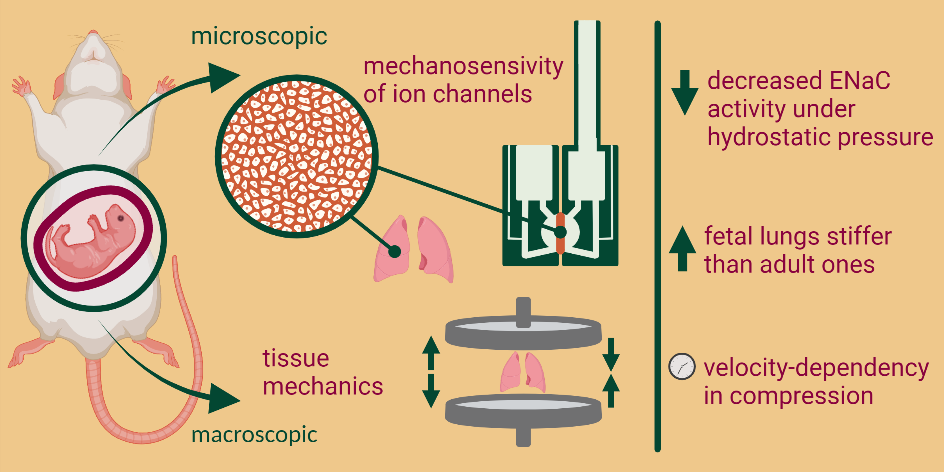

Supplement: Supplementary file 1 [file Image1.TIF]
