# Supplementary material for: Mechanical properties of the premature lung: From tissue deformation under load to mechanosensitivity of alveolar cells
Source: Front Bioeng Biotechnol. 2022 Sep 16;10:964318. doi: 10.3389/fbioe.2022.964318 (PMC9523442; doi:10.3389/fbioe.2022.964318)
Supplement: Supplementary file 2 [file DataSheet1.docx]

Supplementary Material

**Supplementary Figure 1.** Stress-strain curves obtained from tension experiments of fetal rat lung tissue samples with probing velocity of 7 mm·min^-1^.

**Supplementary Figure 2.** Stress-strain curves obtained from compression tests of fetal rat lung tissue samples with probing velocity 7 mm·min^-1^.

**Supplementary Figure 3.** Boxplot of Young’s moduli determined from compression and tension tests of rinsed (w/o) and unrinsed adult rat lung tissue samples with a probing velocity of 7 mm min^-1^. Circles show mean values, while central lines represent medians (50th percentile); the bottom and the top of the box (hinges) represent 1st and 3rd quartiles; the end of the whiskers represent the minimum and the maximum of the data (hinges ±1.5 interquartile range). Statistical significance between groups is marked with *: p < 0.05, **: p < 0.01, ***: p < 0.001.

**Supplementary Table 1.** Number of samples measured in the individual tests.

| Age | Test | Velocity [mm·min^-1^] | Number of samples |
| --- | --- | --- | --- |
| Fetal | **Compression** | **1** | 19 |
|  |  | **7** | 15 |
|  |  | **70** | 18 |
|  | **Tension** | **1** | 18 |
|  |  | **7** | 17 |
|  |  | **70** | 16 |
| Adult | **Compression** | **7** | 8 |
|  | **Tension** | **7** | 8 |
| Adult (rinsed) | **Compression** | **7** | 8 |
|  | **Tension** | **7** | 11 |

**Supplementary Table 2.** P values obtained in the statistical analysis.

| Compared groups | Linear Regression | | Van der Waals model | |
| --- | --- | --- | --- | --- |
|  | **Compression** | **Tension** | **Compression** | **Tension** |
| Adult – w/o | 0.037778 | 0.793122 | 0.00028 | 0.003539 |
| Adult – Fetal 7 mm·min^-1^ | 0.00001 | 0.000195 | 0.000007 | 0.000146 |
| w/o – Fetal 7 mm·min^-1^ | 0.000119 | 0.000177 | 0.000157 | 0.000014 |
| Fetal 1 mm·min^-1^ – Fetal 7 mm·min^-1^ | 0.134548 | 0.900107 | 0.004855 | 0.576985 |
| Fetal 1 mm·min^-1^ – Fetal 70 mm·min^-1^ | 0.000578 | 0.377804 | 0.004882 | 0.359193 |
| Fetal 7 mm·min^-1^ – Fetal 70 mm·min^-1^ | 0.015537 | 0.315462 | 0.049268 | 0.052835 |
